# Defining What is Good: Pluralism and Healthcare Quality

**DOI:** 10.1353/ken.2019.0030

**Published:** 2019-12

**Authors:** Polly Mitchell, Alan Cribb, Vikki A. Entwistle

## Abstract

'Quality' is a widely invoked concept in healthcare, which broadly captures how good or bad a healthcare service is. While quality has long been thought to be multidimensional, and thus constitutively plural, we suggest that quality is also plural in a further sense, namely that different conceptions of quality are appropriately invoked in different contexts, for different purposes. Conceptual diversity in the definition and specification of quality in healthcare is, we argue, not only inevitable but also valuable. To treat one conception of healthcare quality as universally definitive of good healthcare unjustifiably constrains the ways in which healthcare can be understood to be better or worse. This indicates that there are limits to the extent to which improvement activities should be coordinated or standardized across the healthcare sector. While there are good reasons to advocate greater coordination in healthcare improvement activities, harmonization efforts should not advance conceptual uniformity about quality.

## 1.INTRODUCTION

'Quality' is a widely invoked concept in healthcare, and 'quality improvement' is now a central part of healthcare service delivery. However, these concepts and their associated practices represent relatively uncharted territory for applied philosophy and bioethics. In this paper, we explore some of the conceptual complexity of quality in healthcare and argue that quality is best understood to be conceptually plural. Quality is widely agreed to be multidimensional and as such *constitutively plural*. However, we argue that quality is plural in two further senses. First, quality is *competitively plural* : that is, different high-level conceptions of quality can be appropriately invoked in different contexts and serve different purposes. Second, quality is *operationally plural* : the same high-level conception of quality[Other P-367] can be justifiably operationalized differently in different contexts. We argue that this wide conceptual diversity in the definition, specification, and measurement of quality in healthcare is not only inevitable, but also valuable.

Our pluralist account of quality suggests that there are limits to the view, advocated in the healthcare improvement literature, that quality improvement activities should be better coordinated across the healthcare sector (Dixon-Woods and Martin 2016), raising questions about the extent to which such coordination is desirable. Whilst acknowledging the value of greater coordination, we argue that coordination—to the degree that this is taken to involve conceptual uniformity—is not valuable *tout court*. We suggest that any push for conceptual consistency must be accompanied by, and balanced with, an embrace of conceptual diversity. By the end of the paper we will have thus considered some substantive arguments for and against using standardized approaches to defining, specifying, and measuring quality. But beyond this, our account has more deep-seated relevance to bioethics: a core component of our argument is that definitional debates about quality and quality measures only make sense against broader moral assessments of what count as relevant healthcare purposes and good healthcare.

The paper proceeds as follows. In §2, we start to unpack the concepts of 'quality' and 'quality improvement' in healthcare. In §3, we discuss the multidimensionality of quality and argue that definitions of quality are best understood to be heuristic tools, rather than attempts to characterize a single, determinate property of healthcare systems and processes. This indicates that quality is understood to be not just *constitutively plural*, but also *competitively plural*. In §4, we discuss the operationalization of quality in practice, arguing that quality is also *operationally plural*. We illustrate how practicing quality improvement typically involves conceptual interplay between more general definitions of quality and more specified accounts of the indicators and metrics for quality in particular contexts. In §5, we argue that these different forms of contextual variety are valuable and reflect substantive normative differences. We show how this creates challenges for consistency and comparability in healthcare improvement. In §6, we conclude that healthcare improvement should not pursue indiscriminate standardization or seek to erase diversity with respect to quality. Such diversity can reflect thoughtful and justified differences in the conception and operationalization of quality.[Other P-368]

## 2. 'QUALITY' AND 'QUALITY IMPROVEMENT' IN HEALTHCARE

Projects and programs to improve quality form a central part of healthcare delivery. In the UK, every healthcare provider must publish an annual 'quality account,' providing detailed information on its processes and outcomes and responding to a set of questions in order to demonstrate the quality of its services (NHS 2019). Healthcare providers are monitored to ensure that they deliver high quality services (Care Quality Commission 2017). The US has a National Strategy for Quality Improvement in Healthcare, which seeks to improve "overall quality," improve "the health of the population," and reduce the cost of quality care (Agency for Healthcare Research and Quality 2011). By 2009, eighteen European Union member states had a statutory legal requirement for healthcare organizations to have improvement systems (Spencer and Walshe 2009). Because of the significant role that they play in the design and delivery of healthcare services internationally, quality and quality improvement have considerable social and ethical impact. Different ways of understanding, defining, and measuring quality-related concepts and their role in healthcare delivery will result in diverse approaches to service design and, resultingly, different outcomes for patients and citizens. This section will start to flesh out what is meant, or what might be meant, by 'quality' and 'quality improvement' in healthcare.

Quality in healthcare is, broadly, an assessment of how good or bad a healthcare service is, and improving quality is the process of making services better. Assessing the quality of healthcare must therefore start with some account of what quality consists in: what does good (and bad) healthcare look like? Improving the quality of healthcare must begin with some account of what improvement consists in: how can we tell whether one service is better than another? There is, then, an important moral dimension to defining and measuring quality in healthcare, as it involves saying something about what good healthcare and healthcare systems look like and how clinicians, healthcare managers, and policy-makers ought to act. However, 'quality' and 'quality improvement' have taken on somewhat narrower and more technical definitions than these broad evaluative conceptions suggest. There are two main aspects to this. First, the appraisal of quality in healthcare is taken to require a particular kind of evidence. Second, quality improvement practice has become associated with a set of distinctive improvement techniques.

The methodological, evidence-based assessment of the quality of healthcare goes back, at least, to Florence Nightingale's pioneering work[Other P-369] in the Crimean War demonstrating the (positive) relationship between hospital admission and mortality and her efforts, following this, to develop a standardized system of classifying diseases and to systematically track mortality rates (Maxwell 1984). But the 'quality movement' in healthcare took off in the second half of the twentieth century, when scholars and clinicians started to measure systematically deficiencies in medical care, including iatrogenic harms, the use of unnecessary and ineffective medical procedures, and geographical variation (Berwick 2008). Central to this approach—ideally, at least, if not always in practice—is the use of robust evidence to justify claims that interventions have led to improvements in quality (Marshall, Pronovost, and Dixon-Woods 2013). While there is not a single, agreed way to characterize robust evidence, it will likely be obtained using measures that are valid—that is, they measure what they claim to measure—and reliable—they are consistent and reproducible (Pringle, Wilson, and Grol 2002). Evidence-based quality improvement practice also seeks to draw on the full range of evidence, rather than, for instance, basing decisions on the most easily obtainable data or, worse still, cherry-picking findings to suit personal or professional interests (Carter 2018). A commitment to evidence-based quality improvement does not necessarily imply the quantifiability of quality, but numerical assessments of demographics, processes, and outcomes are commonplace as a way of securing justification for claims of good or bad practice.

The practice of quality improvement as a distinctive set of techniques or processes has its roots in the early twentieth century quality movement in industry, which sought to control manufacturing processes in order to reduce variation, eliminate waste, and improve productivity (Junghans 2018). We will refer to the technical practice of quality improvement as QI, to distinguish it from other practices that seek to improve the quality of healthcare. A number of different QI methods grew out of this movement—approaches or tools designed to measure and evaluate current practice and systematically evaluate interventions designed to improve upon it. These include *Plan-Do-Study-Act (PDSA) cycles*, which use short cycles to implement and learn from interventions; *Six Sigma*, a systematic means of assessing the effectiveness of interventions; and *Lean* thinking, a set of tools aimed at reducing waste (Boaden 2009). These methods have been adopted in the healthcare improvement context as means of systematically evaluating healthcare practice and changes to practice. The use of these systematic QI methods, alongside an evidence-based approach, is taken to give improvement practice a certain degree[Other P-370] of scientific rigor and credibility: interventions and their outcomes are assessed and recorded in a methodical and repeatable fashion (Marshall, Pronovost, and Dixon-Woods 2013).

Some QI practice focuses on the reduction of variation in outcomes. In these instances variation is taken to be, in itself, an indicator of avoidable harm to patients and deficiencies in health service delivery (Marshall, Pronovost, and Dixon-Woods 2013). The view that variation is *prima facie* undesirable takes the best outcomes to represent what it is possible for a health system to achieve and worse outcomes to represent avoidable deviation from that peak (Institute of Medicine, Committee on Quality of Health Care in America 2001). Achieving quality in healthcare means minimizing the gap between actual practice in any instance and best possible practice. Identifying some outcomes as better and others as worse requires some account of what good and bad healthcare look like. This will involve reference to the goals or purposes of healthcare, and an assessment as to which practices further these goals and which impede them. These QI practices are, therefore, underpinned by evaluative claims or assumptions about healthcare quality. The gap-closing conception of QI is not ubiquitous. A noteworthy alternative borrows from complexity science and argues that healthcare systems are complex, adaptive systems, within which a certain amount of variation in practice and outcome is not only unavoidable but also valuable and necessary for innovation (Plsek 2001; Plsek and Greenhalgh 2001; Plsek and Wilson 2001; Wilson and Holt 2001). Such complexity models operate with a distinction between unavoidable, expected variation and avoidable variation that ought to be reduced, such as that resulting from error or waste (Plsek 2001). Again, some prior conception of the constituents of good or bad healthcare is needed to give content to these notions of waste and error. So complexity-inspired QI practices are also underpinned by evaluative conceptions of healthcare quality. Without some idea of what would be good or bad healthcare, or of what function healthcare institutions are supposed to play, concepts such as efficiency and appropriate practice remain empty.

The evidence-based conception of quality is now well established and pretty much ubiquitous, and QI is largely seen as a scientific practice, built around a set of techniques (Health Foundation 2011). However, as we have indicated, technical QI practices operate with a prior notion of what constitutes good and bad healthcare. Without this, there is no way of distinguishing evidence that is relevant to assessments of healthcare quality from evidence that is irrelevant. The more technical conception[Other P-371] of QI leans upon a less technical and more straightforwardly normative account of good and bad healthcare and a specification of the goals and purposes of healthcare. In the next section, we explore some of the normative definitions of quality that have been proposed and adopted for use in healthcare improvement practice.

## 3. QUALITY AND MULTIDIMENSIONALITY

It is widely agreed that there are several different dimensions to quality, reflecting a range of purposes and goods that are relevant to healthcare. A number of different accounts of the multidimensional structure of quality in healthcare have been proposed, developed in diverse settings and for different purposes. Multidimensionality, and different accounts of multidimensionality, are key sources of the conceptual pluralism we are investigating. In this section, we briefly review some examples and summarize the significance and implications of multidimensionality. We argue that the specification of multiple dimensions of quality is typically context specific, and it endeavors to pick out the elements of healthcare quality that are salient for a given policy context or healthcare system, rather than to specify a universal definition of quality. Quality, we go on to suggest, is therefore best understood to be not just *constitutively plural*, but also *competitively plural*.

Avedis Donabedian (1978), an early advocate of quality assessment in healthcare, specifies two components to the quality of the performance of individual medical practitioners: *technical* aspects, which reflect the performance of the doctor or healthcare provider, and *interpersonal* aspects, which reflect the relationship between the patient and their doctor. Donabedian (1988) takes different dimensions of quality to be salient depending on the scope of assessment. If the quality of a healthcare institution—rather than an individual doctor—is being assessed, *amenities of care*, which covers factors such as comfort, privacy, and convenience of access, is added to the dimensions of quality. If the quality of the care received by the patient is the object of assessment, the *contribution of patients and family members to care* should also be recognized. If the scope of assessing quality is the care received by a population group, *equality of access to care* becomes salient.

Another early example of a multidimensional concept of quality, developed by Heather Palmer in her discussion of quality in ambulatory care, is not dissimilar from Donabedian's account. Palmer specifies three dimensions: *accessibility*, which reflects the "equitable and timely[Other P-372] distribution of appropriate healthcare to those with equivalent need"; *acceptability*, which captures the "degree to which healthcare satisfies patients"; and *technical competence*, which is the "coordination of knowledge, skill, and judgment in delivering appropriate technology to improve the health of patients" (1988, 120). Other definitions include Robert Maxwell's (1984) six-dimensional concept of quality: *effectiveness, social acceptability, efficiency, access to services, equity* or *fairness*, and *relevance to need*.

The US-based Institute of Medicine (IOM) developed a six-part definition of quality in healthcare, which perhaps remains the most widely used today (Institute of Medicine, Committee on Quality of Health Care in America 2001). Healthcare, it contends, should be *safe, effective, patient-centered, timely, efficient*, and *equitable*. NHS Improvement—the body that is responsible for overseeing UK National Health Service (NHS) Foundation Trusts, NHS Trusts, and independent healthcare providers—develops a concept of quality with four dimensions, which leans on the IOM definition (NHS Improvement 2017): NHS organizations should be *safe, effective, caring*, and *responsive*. Interestingly, NHS Improvement treats *finance and use of resources* and *operational performance* (or timeliness) as separate from quality *per se*, but nonetheless part of good performance of NHS providers.

For the concept of quality in health care to be multidimensional, it must have several different parts that are mutually constitutive of quality. A multidimensional concept of quality is thus *constitutively plural*. The different dimensions make distinctive contributions to an overall assessment of quality. It is possible to show ways in which the dimensions can intersect and inform one another. For example, dimensions involving safety and those relating to clinical effectiveness are likely to be interrelated and mutually constitutive. The same is likely to be true of dimensions concerning equity and those relating to access to services. However, the different dimensions are not reducible one to another. That is, the meaning and value of one dimension cannot be explained in terms of the meaning and value of any of the others; each makes a discrete contribution to quality. Moreover, the multidimensionality of quality implies that quality is not separable from its constituent parts. To improve or worsen along any or all of the dimensions doesn't *lead to* or *cause* an improvement or worsening in quality, it *just is* to improve or worsen quality. This need not necessarily imply that there is nothing that can be said about quality in general or overall, but it does suggest that claims about overall quality must refer to its constitutive dimensions.[Other P-373]

In none of the above proposed conceptions of quality are the dimensions straightforwardly mutually maximizable. They are, to some extent, in tension with one another, such that improving along one axis might introduce constraints along others. This means that the dimensions must sometimes be traded off against one another. So, for example, maximizing safety may constrain timeliness because safety checks and measures to reduce the risk errors and adverse effects can add time to clinical interactions. Improving cost efficiency may constrain clinical effectiveness when additional clinical benefits are deemed not to represent value for money. And clinical effectiveness may constrain patient-centeredness or social acceptability, if achieving the best clinical outcomes fails to represent the patient's wishes or social conventions regarding appropriate care. Some such trade-offs will be deemed reasonable and justified; others will be deemed unreasonable and unjustified. Defining and improving quality involves balancing a range of things we value, which are compatible only in a limited sense.

This is not to say that the dimensions of quality are necessarily or always in tension. Sometimes improving along one dimension will lead to or amount to an improvement along another dimension. So, for example, improvements in clinical effectiveness might lead to better financial efficiency, if they reduce expensive readmissions, iatrogenic harms, or unnecessary care. Indeed, the definitions of the dimensions can overlap, meaning that improving on one dimension—equity, say—creates improvement on another—such as patient-centeredness—by definition. The relationships between the dimensions are complex––sometimes they are in tension, sometimes in agreement––and while they are understood to be distinctive in their contribution to quality, this does not imply that they are entirely mutually exclusive.

The multidimensionality of quality and the tensions between the dimensions together mean that 'quality' is not a single property or attribute of the healthcare system that can be maximized but, rather, an assessment grounded in a number of other attributes.^1^ Healthcare can go well or badly in multiple ways and can produce, or be constitutive of, different combinations and quantities of multiple goods. While it might be possible to make an overall assessment of quality based on assessments of the dimensions, it is unlikely that anything like a simple ranking of quality states could be produced.

The classification of quality into multiple dimensions is typically a pragmatic activity. That is, the dimensional structures are not, and are not[Other P-374] intended to be, exhaustive or universal accounts of the structure and value of healthcare. Each is developed in a context, for a particular purpose, given a set of goals, aims, and values. Some accounts of healthcare quality are explicit about their pragmatism. Donabedian, for example, suggests the following:

The definition of quality may be almost anything anyone wishes it to be, although it is, ordinarily, a reflection of the values and goals current in the medical care system and in the larger society of which it is a part.

(1966, 167)

And similarly, Donabedian argues that "the standards [of good health care] reflect current knowledge and orientations, and are subject to change as knowledge advances and the scope of provider responsibility is redefined" (1968, 182). Maxwell also makes a distinctly pragmatist assertion about the multidimensionality of quality:

The definition of the six dimensions and precisely how many dimensions there ought to be are far less important than the acceptance of multidimensionality, the flavour of the dimensions, and of the policy trade offs among them.

(1992, 174)

Other accounts are more implicit about the context- and purpose-specific nature of their definition of quality. The IOM, for example, writes, "The committee proposes six aims for improvement to address key dimensions in which today's health care system functions at far lower levels than it can and should" (Institute of Medicine, Committee on Quality of Health Care in America 2001, 5). This suggests that the proposed concept of quality is intended to address a particular problem at a particular point in time. NHS Improvement (2017) details a number of practical, specific aims attached to its five themes, which include *quality*, suggesting that their definition is also attached to a specific purpose.

The pragmatic nature of the definitions of quality means that they do not seek to capture everything that could possibly be thought to be important about healthcare. Rather, they aim to specify a set of values that are central to good healthcare in a particular context with a particular set of purposes. Accounts of quality that do no explicitly include something as a dimension do not thereby have to deny its relevance to the quality of healthcare. Some of the definitions discussed highlight ways in which healthcare can be good or bad that are not captured by others. Maxwell's *relevance* dimension captures the importance of assessing the clinical needs of patients in the context of the needs of others in their community; this is not captured by other conceptions. The IOM's *equity* dimension captures[Other P-375] something about the distribution of health and quality of care across a community. NHS Improvement and Donabedian both focus on the quality of the relationship between clinicians and patients, via their *caring* and *interpersonal quality* dimensions respectively, something not explicitly covered in other definitions. Some arguably important values, such as *quality employment* (that is, high standards of employment practices in health services) and *environmental sustainability*, do not feature in any of the mainstream concepts of quality.

Since the definition of quality is a pragmatic matter, 'quality' is therefore a multiply realizable concept. That is, it can be instantiated in a number of different ways depending on the particular context or problem in question. Different multidimensional definitions of quality may reflect different priorities or commitments. This suggests that quality is not only constitutively plural but also plural in a more radical way, which might be deemed *competitively plural*—that is, it is subject to variation, including disagreement, that arises from different vantage points and perspectives.^2^ Competitive pluralism about healthcare quality entails not just that there is reasonable disagreement about the correct definition of quality, but that there are many different conceptions of quality that can be appropriately invoked in different contexts, the specifics of which will depend on the features of particular settings and the contrasting purposes behind the definition and measurement of quality. Different multidimensional frameworks should, thus, be understood to be heuristic tools that enable practitioners and policy-makers to discuss, measure, and assess quality in healthcare in practical settings, rather than attempts to capture the essence of quality or to provide a universal definition. This, moreover, indicates that in order to carry out QI projects, practitioners need to interpret quality for their own purposes and can draw on a very broad range of potential conceptions and dimensions of quality in so doing. This may involve choosing an existing multidimensional framework or adapting an existing framework to suit their purposes and, in some instances, it could involve developing a new framework.

## 4. OPERATIONALIZING QUALITY

Doing QI, at least under the dominant evidence-based paradigm, involves operationalizing whatever conception of quality is under consideration. That is, abstract values such as efficiency, effectiveness, and safety, or other dimensions of quality that are salient in particular instances, must be specified in such a way that it can be determined whether the healthcare[Other P-376] practice under scrutiny embodies them or not. In this section, we argue that the activity of specifying quality for given contexts and purposes corresponds to a third sense in which quality is plural, which we denote *operational pluralism*.

Practicing QI involves making assessments of the quality of a particular healthcare service at different points in time, for example, before and after an intervention has been made. This requires some account of what quality means, specifically, in the context in which healthcare quality is being assessed. This is not to say that QI practitioners must first come up with an abstract concept of quality and, subsequently, decide how to define and measure that concept in practice. The appropriate conception of quality for a particular QI project might emerge only from consideration of actual activities and practices. However, practicing QI requires, at some point, detailed specification of the dimensions of quality. There are several aspects to such specification:

i *Weighting and trade-offs*. Specification involves selecting relevant dimensions of quality and determining how to weight them and trade them off against one another. Weighting involves deciding whether any of the dimensions should be prioritized over others—such that it has more influence in assessments of quality—or whether all should be treated as equal contributors to quality. Determining trade-offs between dimensions involves specifying the scope of each dimension vis-à-vis the others. For example, determining the scope of cost effectiveness involves specifying the point at which increased benefits to clinical effectiveness, safety, and patient-centeredness are no longer worth the additional cost. The weighting and trade-offs that are adopted will reflect the priorities, values, and goals of those seeking to assess the quality of healthcare services. Different approaches to weighting and trade-offs will lead to different assessments of quality.
ii *Indicators*. Specification also involves identification of indicators for each of the dimensions. An indicator is a feature of the healthcare service under consideration, the presence of which denotes high or low performance along a given dimension. For example, clinical effectiveness in prescribing might be indicated by conformity with bestpractice guidelines—a high degree of conformity indicates, all other things being equal, greater clinical effectiveness. Timeliness in a hospital emergency department might be indicated by reasonable waiting times for patients. And patient-centeredness in general practice might be indicated by the involvement of patients in all significant decisions about their care. The indicators for each dimension are likely to be quite different depending on the context under consideration; the measures[Other P-377] and practices that are required for an emergency service to be safe are different from those needed in a GP surgery, a hospice, or a maternity ward. Moreover, in a given context, selecting different indicators of quality dimensions will generate different assessments about the realization of the dimensions in the service under consideration, and so different assessments of quality.
iii *Metrics*. Specification requires the identification of metrics for the indicators. In order to determine whether a clinical service exhibits the specified indicators for the dimensions of quality, some standardized means of assessing the service is required. For example, conformity with best-practice guidelines might be measured by determining the percentage of patients discharged on first line treatment. Adherence to reasonable waiting times might be determined by measuring the percentage of patients admitted, discharged, or transferred within a four-hour window. And the extent to which patients are involved in all significant decisions about their care might be measured using an assessment of patient experience via the Picker Institute patient reported experience measure. Measurement need not necessarily involve numerical quantification. However, it must involve at least *comparable* quantification. That is, when two different services, or the same service at different points, are compared, it must be determinable which has more or less of the relevant indicator (or if they are equal in this respect), even if it there is no way of giving an exact amount by which one is higher than the other. Without some way of comparing different possible states in which a service could be with respect to the indicators, there will be no way of reliably determining its achievement of the determinants of quality (Pringle, Wilson, and Grol 2002).
iv *Data sources*. Finally, specification will involve the identification of the data source used to determine achievement of the metrics. So, for example, the data source for measuring prescribing rates might be clinical audit records; the data source for the time taken to admit, discharge, or transfer a patient might be individual patient records; and the data source for assessments of patient experience might be the results of a recent practice-wide patient experience survey. Selecting the appropriate data source will likely be a largely pragmatic matter, depending on time, resources, past data collection practices and projects, infrastructure, technological equipment, expertise, and so on.

The way in which weighting, trade-offs, indicators, metrics, and data sources are considered, assessed, and decided upon can be intentional or inadvertent, and it can be done thoughtfully or carelessly. If it is done thoughtfully it will be well suited to the purposes and priorities of the group[Other P-378] seeking to assess quality. Otherwise, there is a risk of the context-specific nature of quality being overlooked and particular conceptions being taken to represent quality in instances where they are ill-suited. But regardless of whether they are made well or badly, decisions about the weighting of dimensions, and specification of indicators, metrics and data sources are a necessary part of healthcare improvement practice, as any assessment of the quality of healthcare services involves such operationalization of high-level conceptions of quality. Recognition of the ways that quality is operationalized for use in improvement practice indicates a third way in which quality can be thought to be conceptually plural. That is, the same multi-dimensional quality framework can be operationalized differently, in different contexts, and given different improvement-related aims and purposes. We call this *operational pluralism*.

Quality, then, is conceptually plural in a number of different ways, of which we have highlighted three.^3^ Practicing healthcare improvement requires focus and prioritization. Whether at a system level or in a specific healthcare context, it is necessary to select some combination of concerns as the focus of effort. At both a system level and a local level, improvement priorities will typically arise from practical judgments about where things could be done better. Such judgments might be informed by particular problems or failures that have been recorded, or by documented variation in outcomes. Improvement practitioners will need to identify and prioritize a set of quality-oriented measures and interventions in order to resolve these issues. In each case, 'quality' will be translated into some specific set of dimensions and indicators, reflecting context-specific concerns. Up to a point it is possible to work in parallel on different sets of concerns but there are practical limits to this. All this has the potential to magnify conceptual diversity and lead to a fragmentation of quality assessment: if different concepts, weightings, definitions, and measures are used in different contexts, different—and incommensurable—assessments of quality will result. The assessments will be incommensurable to the extent that they are grounded in different definitions and a different specification of quality, and thus to the extent to which they are simply considering different things. The remainder of this paper considers how far such fragmentation should be resisted or welcomed.

## 5. THE VALUE OF DIVERSITY

In their 2016 paper, "Does Quality Improvement Improve Quality?" Mary Dixon-Woods and Graham Martin highlight the need to look beyond[Other P-379] small-scale QI projects and to "act like a sector" in order to improve the quality of quality improvement (2016, 193). This, they suggest, involves greater coordination of QI activities. Dixon-Woods and Martin highlight the advantages of a sector-wide approach to QI. In particular, sector-wide thinking helps to limit inefficiencies whereby different teams develop localized solutions to the same problem and fail to share their findings with others. A localist approach does not represent good use of resources and may cultivate routines and processes that are only applicable in certain contexts. Such practices encourage what they call "projectness"—"a sense that QI is a series of bounded, time-limited events, rather than a continuous commitment, and overly focused on 'innovation' rather than replication" (Dixon-Woods and Martin 2016, 192). Dixon-Woods and Peter Pronovost (2016) argue that local uncoordinated interventions can have unintended consequences, leading to worse outcomes overall, despite fixing a particular problem at a local level.

To some extent, variation in QI practice is inevitable, and this is recognized by proponents of the sector-wide approach. In order for the same intervention to work in different demographic, institutional, and geographic circumstances, different local variations will need to be modeled and developed. However, for a sector-wide approach, these different variations should contain the same "core, non-negotiable elements" (Dixon-Woods and Martin 2016, 193). One reading of this could be that, while different contexts might call for slightly different local solutions, the goals and purposes of improvement and the definition of quality should be consistent across contexts. This need for some core stability in quality conceptions is captured by the concern that local QI projects may undermine quality overall if not coordinated with respect to the system-wide goals of quality.

Underpinning these arguments for coordination are very important considerations that do not depend upon the claim that QI efforts should be sector wide. If QI initiatives focused on the same set of quality concerns are to be directly comparable across any settings—even just across a couple of institutions—then there is some need for consistency in the way quality is interpreted. This degree of consistency facilitates system-level comparisons of cross-institutional quality measures. It also increases the chance of accumulating the kinds of data that are typically sought within evidence-based QI, data that enable QI practitioners to learn both at scale and from one another's efforts, and to coordinate the efficient use of constrained, and often shared, resources. In many cases, in order to[Other P-380] comprehend how good healthcare is in one context, it will be necessary to compare measures of its processes and outcomes with other relevant contexts. These concerns suggest that there is a limit to the degree to which diversity of quality conceptions is practically desirable. When there is good reason to compare healthcare practice across settings, there will be reason to coordinate quality conceptions.

A full-blown version of a sector-wide approach could require that some overarching conception of quality must be shared by the system as a whole for QI to effectively improve quality. An extreme version might even assert that all QI projects should be centralized. But more moderate versions might permit diversity across local QI projects on the condition that they reflect shared, system-level goals, in order to avoid conflicts relating to the definition and specification of quality. In all cases, any move towards standardization of QI would require some agreement upon a clear and distinct definition of quality in healthcare. This, in turn, would require substantial agreement about and specification of the goals of healthcare.

However, adopting such an approach to QI simultaneously constrains diversity. Establishing a single conception of quality across a system, or even across two or more settings, prevents different priorities from being pursued locally. Because of the complex interaction between the dimensions in a multidimensional concept of quality, adding or removing a dimension from the structure will have implications for the remaining dimensions. A dimension cannot be added or prioritized at a local level whilst maintaining the same weighting and trade-offs between other dimensions. This means that only when QI projects use the same overall definition of quality will they be measuring the same thing and will their assessments be strictly commensurable. Even if two different conceptions of quality include the same dimension—safety, for example—their respective assessments of safety cannot be assumed to be capturing the same thing unless their conceptions of quality also share the same overall structure. Furthermore, quality must be consistently operationalized in order for measures across different settings to be comparable. Therefore, for a local QI project to measure and improve healthcare quality in terms that can be accepted as such by any other QI initiative, the two must use the same definition of quality—with the same dimensional structure and the same specification of the dimensions. Moreover, using one legitimate conception of quality to assess QI practice that is based on another legitimate but divergent conception of quality is to employ an inappropriate standard of assessment. Much hangs, then, on the legitimacy of divergent conceptions of quality.[Other P-381]

As discussed above in §3, different attempts to define quality in healthcare do not typically seek to settle on a universal or final characterization, but rather try to develop a definition that is appropriate for a given purpose. Any particular definition of quality, even those intended for system-wide use, and however well developed, will only provide a partial and context-limited concept. The complexity of QI as a practice stems, in part, from the open-ended and plural nature of the purposes and goals of healthcare (Greig, Entwistle, and Beech 2012). And consequently, working with a single definition of quality is liable to obscure some of the legitimately diverse goals which different actors have and is likely to overlook some of the complexity of healthcare quality. QI actors who are differently positioned will have good reasons to prioritize different conceptions of quality by emphasizing different dimensions, specifying those dimensions differently, and operating with different thresholds and targets of success. These reasons will reflect the diverse roles and responsibilities of actors, different judgments about feasibility, pragmatic factors such as the presence or absence of resources, and different judgments about what combinations of relevant goods matter most. Local definitions of quality will also be shaped by the contingencies of shifting policy and institutional expectations and norms.

Advocating standardized definitions of quality implicitly endorses a rather *technicist* approach, whereby improving quality is a matter of working out the most efficient way to achieve some pre-agreed end. But the choice of quality conceptions, including the tailoring of conceptions undertaken by differently positioned QI actors, is an inherently normative business. This means that it can only ever be a technical process in part. It involves choosing out of the many possible ways of defining and measuring quality, and recognizing that different approaches will lead to different assessments of quality. These choices, in turn, imply different accounts of what good and bad healthcare looks like. This is true both with respect to the broad definition and the definition of dimensions of quality, but also with respect to more detailed aspects of the specification of quality. Planning or evaluating QI adequately involves recognizing that ideas of quality are plural and contested, reflecting diverse and sometimes competing accounts of what we want from our healthcare institutions and practices. Settling on a definition of quality, in broad and specific terms, does not, then, involve determining what quality in healthcare 'in fact' involves so much as thinking carefully about what good healthcare would look like in *this* context. This may, in turn, require consideration of shared[Other P-382] social and political values, as well as reflection on the nature of health and its place within a broader social and personal context. This value-laden decision-making is central to decision-making about quality; unless such reasoning is made explicit, the values underpinning QI practice will be obscure and may lack justification (Carter 2018).

Of course, not all candidate definitions of quality are legitimate. Merely saying that something is an example of good quality healthcare doesn't make it so. However, mere contradiction with other conceptions of quality should not alone discount a new conception. Additional reasons are needed to think that one of the alternatives is better suited to the context or system in question. This indicates that the concern—highlighted by advocates of the sector-wide approach—that local interventions can have unintended consequences that lead to worse outcomes overall needs to be considered in context. Clearly some local interventions may have consequences which conflict with system-level conceptions of quality, but these may not always be unintended, and they may be justified.

This might appear to 'pass the buck' onto procedural considerations. That is, in absence of a substantive solution to the definition and specification of quality, a set of procedural standards is needed to determine, or at least to indicate, how to settle on an appropriate, context-specific account of quality and how to determine whether an existing account of quality is appropriate or not. A full discussion of adequate decision-making processes about the definition of quality in healthcare is beyond the scope of this paper, but we will finish this section with a brief reflection on these questions, which we intend to pick up in future enquiry.

The arguments presented in this paper suggest that the search for a set of procedural standards for appropriate decision-making about healthcare quality cannot sidestep the context-and-purpose-specific nature of healthcare quality. The different contexts in which decision-making about quality arises will be characterized by different decision-making timescales, varying access to data and information, different stakeholder groups to which decision-makers are accountable, and different degrees of impact of decision-making outcomes. All of these, along with many other factors, will affect the kind of decision-making procedures that are appropriate in each case. For example, projects that affect larger numbers of people, involve multiple providers, or involve decisions with serious human consequences, are likely to involve higher standards of evidence, greater justification, and wider consultation of stakeholders than more localized, less consequential projects. The same factors that make it[Other P-383] necessary to use different definitions of quality in different contexts also, therefore, make it necessary to use different decision-making procedures in relation to defining quality in different contexts. Extending pluralism to the procedural domain is crucial because what is at stake here is not merely reasonable pluralism about the definition of quality, but reasonable pluralism about indefinitely many context-specific definitions of quality. The task of defining healthcare quality is one that arises repeatedly, in different contexts, and a procedural solution that is appropriate in one context need not be appropriate in others.

This does not mean that nothing whatsoever can be said about such decision-making procedures. Assessing the legitimacy of different conceptions of quality will likely involve determining whether the people who developed it thought carefully about the set of problems with which they were presented and whether they had good reasons for prioritizing some issues over others. It will typically require deliberation about the goals and purposes, implicit and explicit, of the healthcare system in question and of those who seek to assess and improve it. It might also involve consideration of the extent to which their definition secures agreement within the relevant community of stakeholders—the public, patients, commissioners, staff, and so on. However, what these stipulations involve, and the different standards of evidence, argument, and justification that are involved in their execution, are likely to be quite different in different contexts.

## 6. BALANCING STANDARDIZATION AND DIVERSITY

There is, then, plenty of room for legitimate disagreement about which aspects of quality should be emphasized in general and in specific cases and which conception of quality should be used. Although there are good arguments for forms and degrees of coordination between QI initiatives, coordination should not automatically be taken to entail consistency of definitions, but might sometimes mean clear mapping of the differences between definitions. There are advantages to a sector-wide approach insofar as it involves envisaging an overall picture of large-scale quality agendas, and this may support some elements of standardization, but there are limits to how far this standardization is desirable. Insofar as coordination across QI projects is important, such an approach needs to exhibit a reflexive awareness of the normative nature of quality conceptions and recognize the need to specify and balance different quality concerns in local contexts, at a system level, and between local and system levels. This[Other P-384] can be done more or less self-consciously and it is important to consider how we could do it well, or at least better. While standardization about the definition of healthcare quality is valuable in relation to given ends and purposes, it is not valuable for its own sake, and therefore justification for standardization and coordination needs to be sought in order to ensure that it does not stifle legitimate variation in quality conceptions.

Some QI responsibilities are relatively local––for example, those focused on improving the safety of specific local services––while others are much broader and will relate to institutions or even systems as a whole. In both cases there is a need to be aware of coordination challenges so as to avoid some of the limitations of 'projectness,' but system-level roles must clearly have a particular regard to quality coordination issues—they must pay regard to the range of relevant quality dimensions and to the dangers of well-intentioned efforts in one quality domain or part of the system undermining concerns in other quality domains or parts of the system. There is, doubtless, much value to be derived from QI projects that take a system-level approach. They allow us to compare practices and outcomes between institutions, to consider geographical factors, and to compare different commissioning contexts. But if the nature of quality is plural and contestable, then the value of coordination is limited. If there is no single correct or best way of improving quality in healthcare and of measuring quality and quality improvement, then standardizing QI efforts in line with pre-defined conceptions of quality will constrain the possible meanings of quality and the ways in which the goals and purposes of healthcare can be beneficially construed. Thus, standardization will always carry costs and require justification, and it will sometimes fail to be justified.

Accordingly, coordination of QI activities should be pursued only insofar as it does not quash legitimate local QI projects, and insofar as the conception of quality that coordinated efforts endorse is in fact appropriate in the contexts in which it is employed. If conceptions of healthcare quality are understood to be heuristic decision-making devices, such that there is no way of determining the best or correct overall characterization of quality, then tension between different accounts of quality can be seen as a predictable and valuable feature of healthcare systems rather than a predicament to be overcome.[Other P-385]

## NOTES

1 It might, of course, turn out that the dimensions of quality are *also* multidimensional and not straightforwardly maximizable.

2 This distinction between constitutive and competitive pluralism is adopted from Amartya Sen's (1987) discussion of assessments of the standard of living.

3 We take 'conceptual pluralism' to be ambiguous as to the sense in which a concept is plural. Depending on the theory of 'concept' that you are operating with, you might take competitive pluralism and operational pluralism to entail that there are many different concepts of quality or to entail that there is one concept of quality which is realized in a variety of ways. We intentionally leave this open here.
